# Exploring microbial diversity responses in agricultural fields: a comparative analysis under pesticide stress and non-stress conditions

**DOI:** 10.3389/fmicb.2023.1271129

**Published:** 2023-10-20

**Authors:** Saurabh Gangola, Samiksha Joshi, Geeta Bhandari, Garima Pant, Anita Sharma, Kahkashan Perveen, Najat A. Bukhari, Ranjana Rani

**Affiliations:** ^1^School of Agriculture, Graphic Era Hill University, Bhimtal, India; ^2^Department of Biosciences, Swami Rama Himalayan University, Dehradun, India; ^3^Department of PDP, Graphic Era Hill University, Bhimtal, India; ^4^Department of Microbiology, GBPUAT, Pantnagar, India; ^5^Department of Botany and Microbiology, College of Science, King Saud University, Riyadh, Saudi Arabia; ^6^School of Agriculture and Food Science, University of Queensland, Brisbane, QLD, Australia

**Keywords:** pesticides, biodegradation, metagenomics, agriculture, environment

## Abstract

Exposure to pesticides changes the microbial community structure in contaminated agricultural fields. To analyze the changes in the native microbial composition qRT-PCR, a metagenomic study was conducted. The qRT-PCR results exhibited that the uncontaminated soil has a higher copy number of 16S rDNA relative to the soil contaminated with pesticide. Metagenome analysis interprets that uncontaminated soil is enriched with proteobacteria in comparison with pesticide-contaminated soil. However, the presence of Actinobacteria, Firmicutes, and Bacteroides was found to be dominant in the pesticide-spiked soil. Additionally, the presence of new phyla such as Chloroflexi, Planctomycetes, and Verrucomicrobia was noted in the pesticide-spiked soil, while Acidobacteria and Crenarchaeota were observed to be extinct. These findings highlight that exposure to pesticides on soil significantly impacts the biological composition of the soil. The abundance of microbial composition under pesticide stress could be of better use for the treatment of biodegradation and bioremediation of pesticides in contaminated environments.

## Introduction

The application of agrochemicals including fertilizers and pesticides has become an essential component of agriculture (Malla et al., 2018). Consumption of pesticides is tremendously increasing globally in order to fulfill the food demand of the growing population (FAO, 2021; Gangola et al., 2023a). The global consumption of pesticides was 4.2 metric tons in 2021 and is expected to reach 4.4 million metric tons by 2026, with a 0.5% increase every year (PMO, 2022). Based on the consumption of pesticides, China is at the top, followed by the United States, Brazil, and Argentina. Pesticides can travel from the soil surface to a water reservoir or groundwater, and their fate depends on the environmental conditions, such as adsorption to the matrix/soil sediment, transport with water, chemical transformation, and formation of recalcitrant metabolites (Gonzalez-Rodriguez et al., 2011). Although the formation of pesticides is examined under standard rules and systems, some are very recalcitrant in nature, becoming a threat to the ecosystem and polluting water bodies (Sjerps et al., 2019).

Agricultural and natural habitats are regularly contaminated by anthropogenic activity (Jeffries et al., 2018). The application of pesticides on agricultural fields released into the environment and reaching the soil surface, different water sources, and underground water is of major concern for environmental sustainability and human health (Aldas-Vargas et al., 2022). Transformation of pesticides in the environment depends on environmental conditions and physical, chemical, and biological degradation mechanisms (Gangola et al., 2023b). Microorganisms play a significant contribution in nutrient recycling, enhance crop growth and nutritional quality, and are vital components of our living environment. Therefore, it is necessary to check the negative impact of pesticides on beneficial microbial populations and their surrounding environment (Gangola et al., 2022a; Bhatt et al., 2023). The implementation of a microbial system for the degradation of xenobiotic compounds from a contaminated environment is the most favorable approach for the sustainable environment and human health (Saibu et al., 2020; Doolotkeldieva et al., 2021). The complete dissolution of pollutants from contaminated sites depends on several factors such as concentration, chemical structure, temperature, pH, soil microbial community composition, and their activity (Kowalczyk et al., 2015). Due to the variation in season, geographical location, and environmental conditions, the distribution of pesticides is uneven and affects microbial composition significantly (Verma et al., 2013; Raj et al., 2023). The process of biodegradation may vary from one ecosystem to another and depends more on the microbial composition as agricultural soil is rich and active in microbial composition as compared with the oligotrophic environment. Hence, monitoring pesticide biodegradation mechanism by structural and functional attributes of native microflora under different environmental conditions and their environmental fate is important as an indicator and for better understanding of the study (Fenner et al., 2013). Microorganism-mediated pesticide mineralization involves several chemical reactions such as oxidation–reduction, dehalogenation, hydrolysis, dehydrogenation, dealkylation, methylation, conjugation, and ring cleavage (Cycoń et al., 2017). Additionally, the development of a novel approach is crucial to describe microbial diversity and give in-depth knowledge of microbial responses to pesticide exposure (Gangola et al., 2022b,c).

Pesticide concentration, residue, and metabolites are the traditional indicators that are not applicable for several pesticides in an anaerobic environment. Moreover, these indicators are unable to differentiate between biotic and abiotic pesticide biodegradation processes (Aldas-Vargas et al., 2022). Therefore, the use of advanced research tools is important to monitor genes involved in the biodegradation of pesticides or identify the microorganisms in the contaminated environment. The cultivation-dependent approach only allows to cultivate a small proportion of total microorganisms and restricts their accessibility for research study (Schloss and Handelsman, 2006). However, in cultivation-independent methods, the study relies on DNA sequencing of the environmental sample to examine the complete study of microbial community structure, biomass composition, nutritional status, and physiological stress response for a particular environment (Su et al., 2012; Costa et al., 2020). The introduction of advanced technology such as metagenomics and metabolomics is under development and has shown their promising application in characterizing pesticide effects on soil biomass (Hou et al., 2015; Jeffries et al., 2018; Malla et al., 2022).

From the development of next-generation sequencing (NGS), the researchers preferably work on targeted and non-targeted genes to explore more information using some advanced techniques such as metagenomics and metatranscriptomics. These molecular tools have enough potential to extract the entire microbial composition and their metabolic potential without having any prior knowledge (Zhou et al., 2015). The metagenomics-based approach extends several new ways of opportunities to explore the dominant pesticide-degrading genes and their distribution in different microbial genera both in culturable and in non-culturable microorganisms within a complex environment (Fang et al., 2018). Aldas-Vargas et al. (2022) used a metagenomic approach to monitor the biodegradation of pesticides. Through implementing metagenomics, the genes *atzABCDEF* responsible for atrazine biodegradation were identified in agricultural soil (Malla et al., 2022) and the rhizospheric region of different trees (Aguiar et al., 2020). The metagenomic approach was successfully used to study seasonal variation in microbial communities and pesticide biodegrading genes linked to metabolic pathways from different aquatic environments such as freshwater and marine sediments (Fang et al., 2014). These water bodies were contaminated with 10 pesticides, namely, atrazine, carbendazim, chlorothalonil, isoproturon, linuron, metamitron, nicosulfuron, 2,4-dichlorophenoxyacetic acid (2,4-D), organophosphates, and pyrethroid (Fang et al., 2014). After metagenomic analysis of the activated sludge sample, a total of 68 subtypes of pesticide-degrading genes were identified, and out of them, *dhn* gene (encode dehydrogenase and degrade metamitron) was found to be dominant. Pesticide contaminates the metagenomic analysis of soil sample, revealing that as the concentration of pesticide increases in the soil, the expression and number of pesticide-biodegrading genes also increases and are mostly peroxidase, monooxydase, and cytochrome P450 (Russell et al., 2021). Hence, these studies confirmed that high-throughput techniques have enough potential to examine the microbial community and their different powerful pesticide-degrading genes under complex environments. Very limited study has been conducted on the comparative microbial community analysis of pesticide-contaminated and non-contaminated soil. Therefore, this study aimed to analyze and differentiate the microbial community of pesticide-contaminated and non-contaminated agricultural soil.

## Materials and methods

Soil samples were collected from two different agricultural fields in Gularbhoj (29.0918°N, 79.3156°E), Uttarakhand, India. One field was contaminated with pesticides, and the other was uncontaminated. The rice crop was grown in both fields during the Kharif season. The soil sample was collected using stainless steel auger. The 10 cm of soil at the top was discarded, and the next 5 cm of soil was collected from both the contaminated and uncontaminated sites. Both the soil samples were labeled and stored in a deep freezer at −20°C. The soil sample taken from uncontaminated sites acts as a control for this study. Pesticide residues such as chlorpyrifos, cypermethrin fipronil, and imidacloprid were majorly found in the pesticide-contaminated soil. Soil DNA was extracted from both soil samples (500 mg) using the HiPurATM Soil DNA Purification Kit. The purity was checked using a NanoDrop spectrophotometer at wavelengths of 260 and 280 nm with a concentration of 50 ng/L (Jeffries et al., 2018; Malla et al., 2022).

The highly variable region (V3–V4) of the 16SrRNA gene in the soil bacterial community was targeted using the Illumina MiSeq platform. The primers used for amplification were V3: 341 F (5′ CCTACGGGAGGCAGCAG 3′) and V4: 806 R (5′ GGACTACHVGGGTWTCTAAT 3′). Paired-end reads obtained from sequencing were subjected to several quality control checks, including score distribution, base quality, average base content, and GC distribution. The FLASH program (Magoč and Salzberg, 2011) was utilized to merge the paired-end reads. High-quality reads ranging from ~350 to 450 base pairs were obtained by applying multiple filters. The UCHIME algorithm was employed to identify and remove chimeric sequences. Subsequent analysis of the data was performed using the QIIME program (version 1.9.1) (Caporaso et al., 2010). The pre-processed reads were pooled and clustered into operational taxonomic units (OTUs) at a 97% similarity threshold using the UCLUST program. Representative sequences for each OTU were chosen by aligning the sequences against the Greengene database *via* PyNAST (DeSantis et al., 2006; Jeffries et al., 2018). Taxonomic classification was conducted using the RDP classifier against the SILVA 16S rRNA gene database (Joshi et al., 2021). Obtained reads and OTUs from both samples were classified into bacterial phylum and genera (Khati et al., 2019). Statistical Analysis of Metagenome Package (STAMP) was used for additional statistical analysis and heatmap visualization, while UPGMA clustering was employed to generate dendrograms (Parks et al., 2014). Alpha diversity was assessed by the Shannon index using the QIIME program (version 1.9.1) (Chaudhary et al., 2021; Joshi et al., 2021).

### qRT-PCR analysis

The 16S rDNA extracted from the soil was subjected to qRT-PCR analysis using the iCycler iQTM Multicolor instrument (Bio-Rad Laboratory, Hercules, CA, USA) and SYBR green dye (Kumar et al., 2019). The qRT-PCR amplification employed a pair of universal primers, specifically primer 1 (5′-CCTACGGGAGGCAGCAG-3′) and primer 2 (5′-ATTACCGCGGCTGCTGG-3′).

## Results

### Real-time PCR analysis

In both the soil samples' native soil bacterial community, their abundance was observed using high-throughput sequencing and qRT-PCR analysis. After qRT-PCR analysis, it was observed that pesticide-contaminated soil had less copy number of 16S rDNA than uncontaminated soil, i.e., 1.96 × 10^8^ and 5.25 × 10^8^, respectively, per gram of soil.

### Comparative microbial diversity analysis

Comparative analysis for efficient functional microbiome and taxonomic community composition under pesticide stress and non-stress conditions was performed with the help of a high-throughput metagenomic approach.

**Figure 1:**
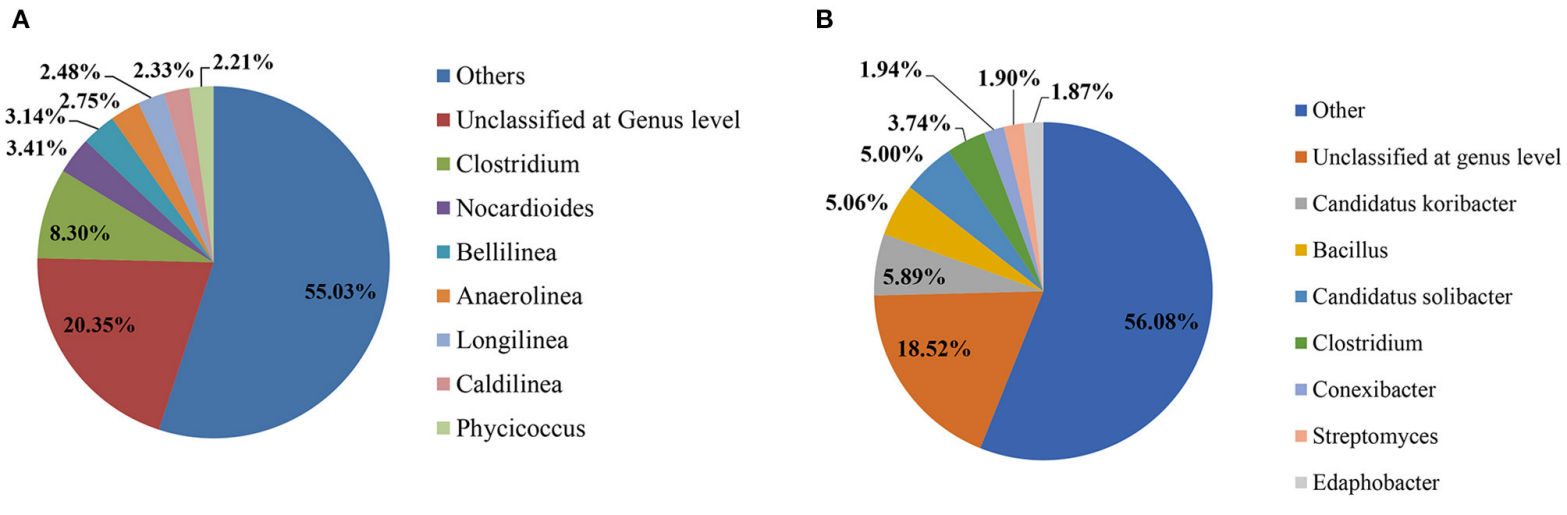
Pie chart provides a comparative analysis of the impact of pesticides on the soil microbial community at the genus level. **(A)** Depicts the soil sample contaminated with pesticides (2G), while **(B)** represents the soil sample without any pesticide contamination (2GC).

Total reads in the pesticide-contaminated (2G) and non-contaminated (2GC) soil samples were 562,416 and 873,083, respectively. Furthermore, the reads were classified at the phylum and genus levels, i.e., for contaminated soil, the reads were 562,416 and 716, while for non-contaminated soil, the reads were 873,083 and 725. During the study, only the top 8 dominant phyla and genera were selected for comparative analysis. At the genus level, the unclassified category comprised of most abundant genera in both the 2G (20.35%) and 2GC (18.52%) soil samples. In the 2G soil sample, the second most dominant genus was *Clostridium* (8.30%), subsequently followed by *Nocardioides* (3.41%), *Bellilinea* (3.14%), *Anaerolinea* (2.75%), *Longilinea* (2.48%), *Caldilinea* (2.33%), and *Phycicoccus* (2.21%). In 2GC soil sample, the second most abundant genus was *Candidatus Koribacter* (5.89%), subsequently followed by *Bacillus* (5.06%), *Candidatus Solibacter* (5.00%), *Clostridium* (3.74%), *Conexibacter* (1.94%), *Streptomyces* (1.90%), and *Edaphobacter* (1.87%) (Figure 1).

Additionally, the comparative study at the phylum level exhibited the prominent existence of Firmicutes (specifically Clostridium), Actinobacteria (Nocardioides and Phycicoccus), and Chloroflexi (Bellilinea, Anaerolinea, Longilinea, and Caldilinea) in the 2G soil sample (Supplementary Figure). However, in the 2GC soil sample, the dominant phylum was Acidobacteria (specifically *Candida tuskoribacter* and *Candida tussolibacter*), followed by Firmicutes (*Bacillus and Clostridium*), Actinobacteria (*Conexibacter and Streptomyces*), and Proteobacteria (*Edaphobacter*). The phyla Proteobacteria, Actinobacteria, Firmicutes, Bacteroidetes, and Planctomycetes were consistently present in both soil samples. Two unique phyla, Chloroflexi and Nitrospira, were found exclusively in the 2G soil sample. With the exception of *Clostridium*, the genus-level composition of the microbial communities exhibited variations between the two soil samples.

### Alpha diversity

**Figure 2:**
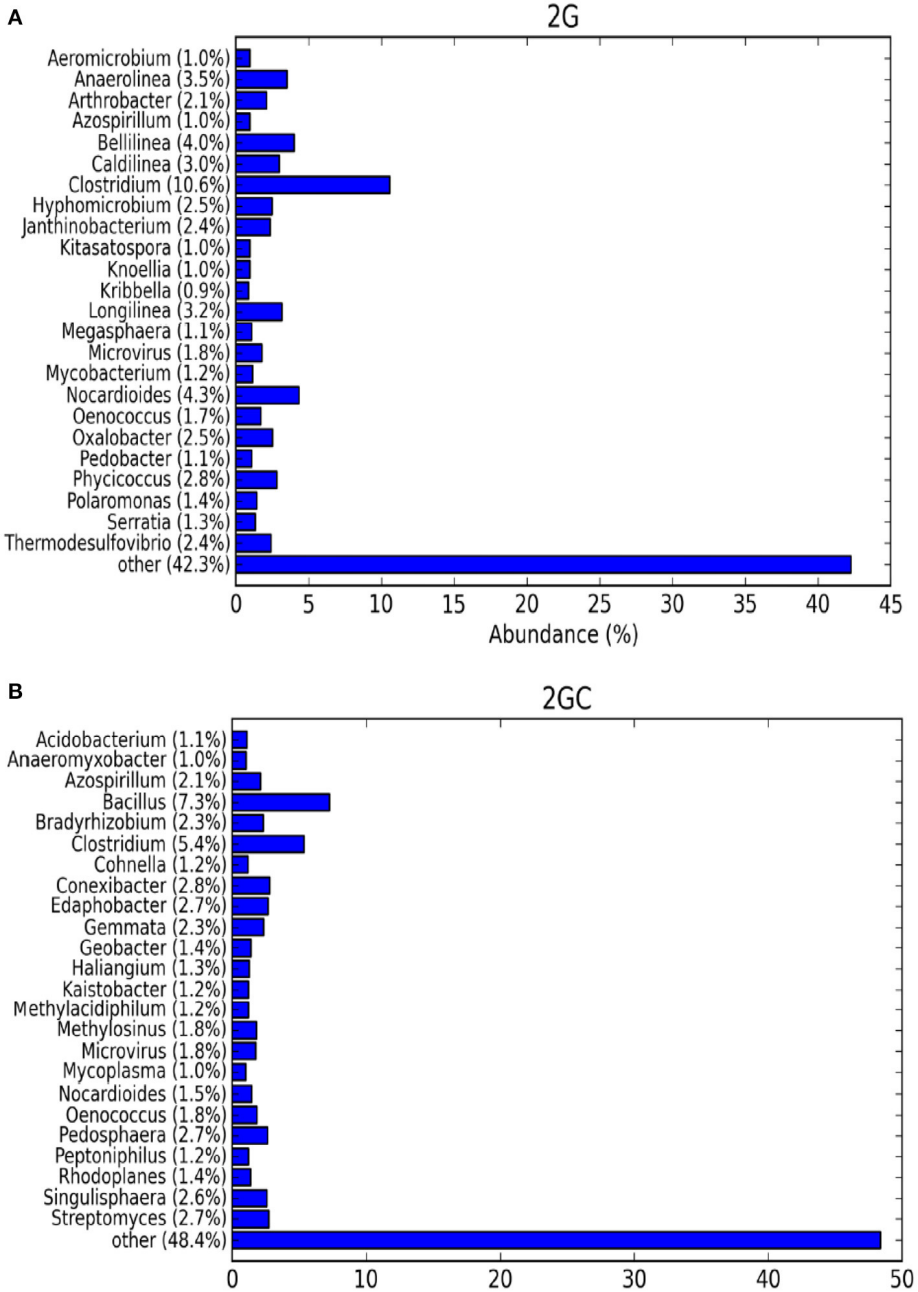
The bar graph exhibits alpha diversity of bacterial communities. The top 25 OTUs, taxonomically classified at genus level of **(A)** pesticide contaminated (2G) and **(B)** non-contaminated (2GC) soil samples.

The genus-level relative abundance of the top 25 classified operational taxonomic units (OTUs) was investigated. In the 2G soil sample, the Shannon species diversity index was high (3.198). Genotypically, a total of 1,627 species were identified, whereas in the 2GC soil sample (control), the Shannon species diversity index was 2.739, and 1,850 species were identified genotypically (Figure 2).

In the 2G soil sample, the population of *Clostridium* was larger, whereas in the 2GC soil sample, the population of *Bacillus* was dominant. Unclassified bacteria at the genus level were predominantly found in both soils, but their abundance decreased in the 2GC soil sample. Genera from the phyla Actinobacteria (*Nocardioides*) and Firmicutes (*Clostridium and Oenococcus*) were evenly distributed in both soil samples. Abundant genera in the 2G soil sample included *Clostridium* (10.6%), *Nocardioides* (4.3%), *Bellilinea* (4.0%), *Anaerolinea* (3.5%), *Longilinea* (3.2%), and *Phycicoccus* (2.8%). The remaining microbial population in the 2G soil sample was distinct from that in the 2GC soil sample.

### Hierarchal clustering

**Figure 3:**
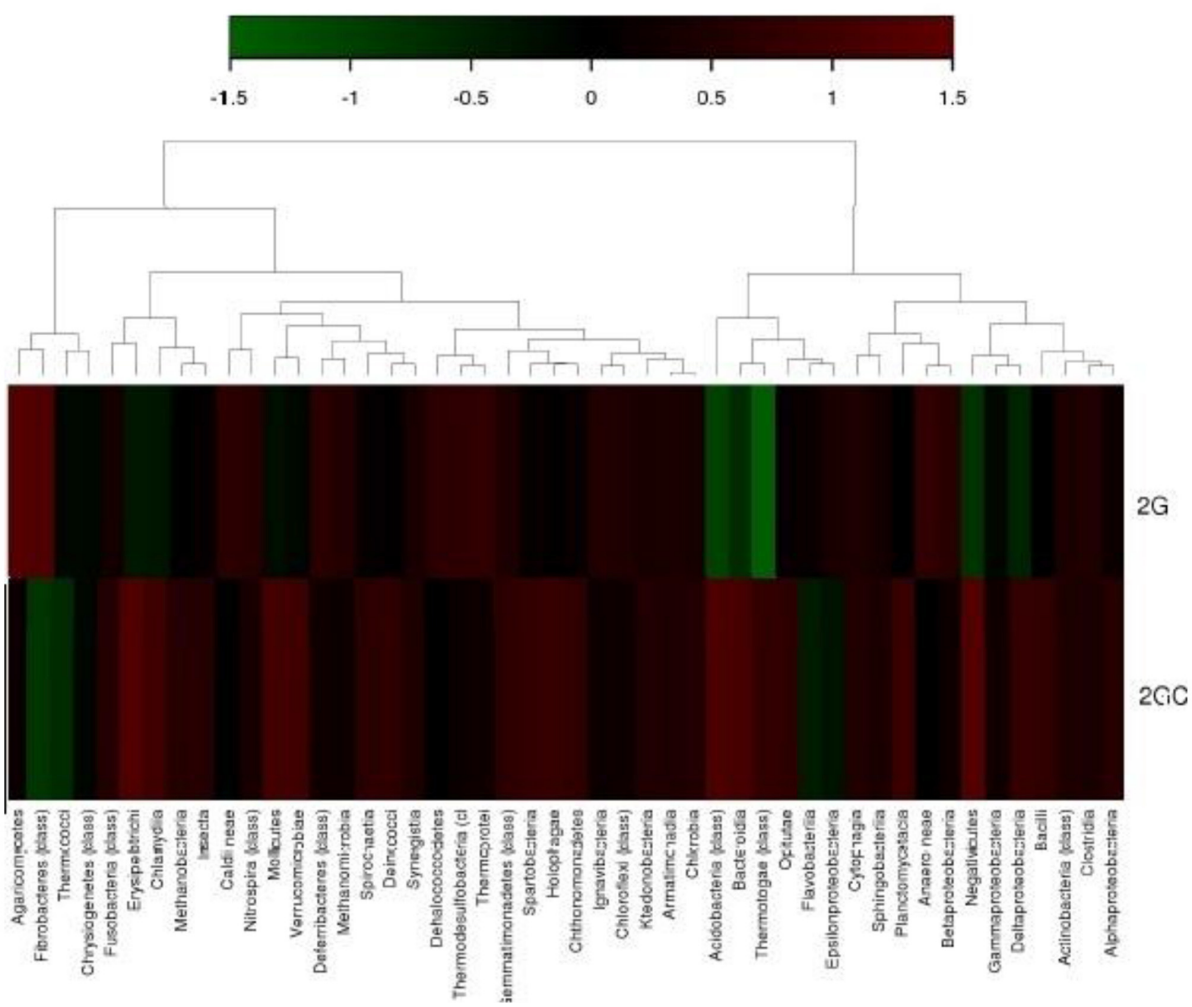
Hierarchical clustering illustrates the total count of operational taxonomic units (OTUs), according to the number of hits and % hits against the Greengene database. The heatmap indicates the abundance of each class within every sample. The color gradient from deep brown (+1) to dark green (−1) represents the distribution of OTUs, ranging from higher to lower abundance.

The heat map generated by hierarchical clustering exhibited the number of OTUs per sample. The color intensity on the heat map corresponds to the relative abundance of an OTU within a sample (Figure 3). In the heat map generated by hierarchical clustering, the pesticide-contaminated soil sample (2G) exhibited significant prevalence of the following classes: Clostridia, Betaproteobacteria, Ignavibacteria, Gemmatimonadetes, Dehalococcoides, Caldineae, and Thermoprotei.

## Discussion

Several microbial communities have been studied previously in response to pesticide contamination (Floch et al., 2011; Zabaloy et al., 2012); however, fewer studies have utilized metagenomics for this purpose. Gangola et al. (2021) isolated a bacterial strain *Bacillus cereus* 2D from the same sampling sites, i.e., Gularbhoj, and found a highly efficient strain to tolerate higher concentrations of pesticides and a high rate of degradation, and they evaluated the expression of protein profiling under pesticide stress. The decrease in the copy number of 16S rDNA genes in the pesticide-contaminated soil indicated exposure of the pesticide to the native microbial flora, leading to inhibition of metabolic activity and growth. Application of real-time PCR was also employed by several researchers for the investigation of gene expression, microbial abundance, and functional and taxonomic gene expressions (Rastogi and Sani, 2011; Gangola et al., 2018; Kumar et al., 2019; Joshi et al., 2021). Yale et al. (2017) used quantitative PCR to assess and quantify the expression of pesticide-degrading genes (trzN, atzB, and atzA). Bacteria play an important role in balancing the ecosystem, nutrient composition, and cycling, but exposure to pesticides suppresses the microbial community significantly in agricultural fields (Onwona-Kwakye et al., 2020). Although the development of molecular techniques (qPCR) is quite laborious and difficult, these techniques give more insights into and better monitoring of pesticide-degrading genes.

Several hidden facts have been resolved since the development of emerging explorative techniques such as metagenomics. However, though it requires extensive data analysis, it justifies its potential in monitoring more than one pesticide compound at a time and analyzing their effects on the microbial community at different levels, such as class, phylum, genus, and species levels (Hou et al., 2015; Aldas-Vargas et al., 2022). The decrease in reads in the pesticide-treated soil (2G) is supported by various findings that clearly indicate the toxic nature of the pesticide (Onwona-Kwakye et al., 2020; Bhatt et al., 2021; Gangola et al., 2022d). The reduction in read numbers in the 2G soil sample may be attributed to the excessive and repeated use of pesticides in agricultural practices, ultimately resulting in the decline in the microbial population. Regular application of different pesticides decreases the microbial communities such as *Aeromonas, Bordetella, Comamonas, Enterobacter*, and *Staphylococcus*. Apart from that, exposure to pesticides may decrease the degradation rate of organic pollutants, microbial homeostasis, and plant growth and protection from pathogens (Hayward et al., 2010; Khalifa et al., 2016; Hamidou Soumana et al., 2017; Pereira et al., 2017). Kantachote et al. (2001) and Rodríguez et al. (2020) have demonstrated the negative impact of pesticides on soil microorganisms on agar plates.

The large portion covered by others in the pie chart represents those microorganisms presenting their existence in a very minor proportion compared with others present dominantly, while the dominance of the unclassified category in both the soil samples represents those microorganisms that are still not identified and classified or are novel. In the 2G soil sample, *Clostridium* is the dominant genus followed by *Nocardioides, Bellilinea, Anaerolinea, Longilinea, Caldilinea*, and *Phycicoccus*. *Clostridium* has already been reported for its pesticide degradation potential such as Alachlor, chlorpropham, DDT, and lindane and is capable of surviving in pesticide-contaminated soil (Alipour et al., 2018). The presence of atrazine and chloro-s-trazine-degrading enzyme TrzN was identified in *Nocardioides* sp. and utilizes atrazine as the sole carbon and nitrogen source from contaminated soil (Topp, 2001; Ortiz-Hernández et al., 2013). Although *Bellilinea* sp. and *Longilinea* sp. have not been reported for pesticide biodegradation, in previous report, *Bellilinea* sp. showed their potential to degrade a carcinogenic xenobiotic compound, i.e., 2-methyl-naphthalene, while *Longilinea* sp. was characterized for propionate degradation (Yamada et al., 2007; Musat et al., 2009; Rodríguez et al., 2020). The role of *Anaerolineae* has been reported to degrade and minimize the concentration of different xenobiotic hydrocarbons under wastewater (Yamada et al., 2007). In soil conditions, the initial application of chlorpyrifos suppresses the expression of genes and metabolic action of the native microbial community; after 2 weeks of the application, the suppressed microbial community reverts back to the level of control (non-contaminated soil) (Fang et al., 2008). Hence, it concluded that due to the different environmental fate of the pesticides after their applications, the native microbial community gets a chance to recover or can adapt to the conditions by expressing pesticide-degrading genes and metabolic potential.

Our research reveals variations in microbial communities and population sizes, signifying the repeated use of pesticides in agricultural fields. This leads to the replacement of older populations with new ones over time. As a consequence, the number of pesticide-sensitive species declines while pesticide-tolerant species survive and proliferate (Gangola et al., 2021). Our findings indicate that pesticides have a toxic impact on specific species within the same phylum, while others are capable of utilizing pesticides as a source of carbon and energy, enabling their prolonged survival. The increased population sizes of Proteobacteria, Actinobacteria, Firmicutes, and Chloroflexi point to their active growth and proliferation in pesticide-contaminated soil. The positive association between these present microbes in consortia may enhance their degradation abilities under pesticide stress (Yilmaz et al., 2022). Furthermore, our study demonstrates that microbial communities with a high metabolic potential for pesticide degradation are more abundant in soils where pesticides persist for an extended period or are regularly used over time. Comparing the two soil types, we observed that the abundance of highly metabolically active communities was higher in rapidly degrading soil, indicating the superior functional capacity of these microbes in terms of nutrient cycling. Consequently, we can infer that the microbial community in 2GC soil exhibits sensitivity to pesticides, whereas the microbial community in 2G soil displays resistance to them (Gangola et al., 2021). At the phylum level, our research indicates that these microbes utilize pesticides as a source of carbon and energy for their growth and development. The toxic nature of pesticides leads to the absence of certain bacterial populations in the treated samples as compared with the control sample (Jeffries et al., 2018). Notably, the higher richness of Firmicutes, Actinobacteria, and Chloroflexi in the treated soil suggests their active involvement in pesticide-contaminated soil.

As observed in the alpha diversity analysis, the Shannon species diversity index (3,198) was maximum for the 2G soil sample, while in the 2GC soil sample, the Shannon species diversity index was minimum (2.739). This indicates that more the Shannon index value, the more the diversity in species. Hence, it could be analyzed easily that the more diversity in the system, the more the system stable. Although the total number of species (1,850) present in 2GC soil is more as compared with the 2G (1,627), in the context of species diversity, 2G soil sample is more dynamic. The possible reason is that pesticides create selective pressure on the microbial community to adapt and acclimatize under stress.

In the hierarchical map, the different classes of microbial communities were analyzed in a comparative manner between 2G and 2GC soil samples. More intense color signifies the dominance of the native microbial class. A hierarchical map was used by Parks et al. (2014) and Jeffries et al. (2018) to analyze the relative abundance of microbial functions in soil samples contaminated with organophosphorus pesticides. Previous reports also marked similar communities for pesticide degradation in different studies. Parks et al. (2014) and Jeffries et al. (2018) employed the hierarchical map approach, utilizing UPGMA clustering, to investigate the comparative prevalence of microbial function in soil samples contaminated with organophosphorus pesticides.

## Conclusion

The application of indigenous microorganisms present in contaminated environments is a more reliable approach for the biodegradation of agricultural pesticides. Under pesticide stress, the bacterial communities altered their genetic pool and developed as efficient mutant strains for the utilization of pesticides as a source of carbon and energy. Monitoring of microbial communities is important because they play a crucial role in the regulation of biogeochemical cycles and soil health, enhancing crop productivity, and maintaining a sustainable environment. Additionally, by conducting metagenomic studies of contaminated soil samples, it becomes possible to identify various types of microbial populations present and the interaction between them. This knowledge is indispensable for comprehending the natural biodegradation of pesticides in environmental settings. Looking ahead, exploring the activity of bacterial isolates against other xenobiotic compounds and delving into the mechanisms of gene regulation involved would be valuable avenues for research and potential advancements in the field.

## Data availability statement

The original contributions presented in the study are publicly available. This data can be found in the NCBI repository under accession numbers: SAMN37722849 and SAMN37722850.

## Author contributions

SG: Conceptualization, Data curation, Formal analysis, Writing—original draft. SJ: Conceptualization, Formal analysis, Writing—review and editing. GB: Formal analysis, Investigation, Methodology, Writing—review and editing. GP: Writing—review and editing, Formal analysis. KP: Formal analysis, Validation, Writing—review and editing. NB: Formal analysis, Validation, Writing—review and editing. RR: Validation, Writing—review and editing. AS: Formal analysis, Supervision, Validation, Writing—review and editing.
