# Supplementary material for: Exploring microbial diversity responses in agricultural fields: a comparative analysis under pesticide stress and non-stress conditions
Source: Front Microbiol. 2023 Oct 20;14:1271129. doi: 10.3389/fmicb.2023.1271129 (PMC10623313; doi:10.3389/fmicb.2023.1271129)
Supplement: Supplementary file 1 [file Data_Sheet_1.docx]

**SUPPLEMENTARY FILE**

**Exploring Microbial Diversity Responses in Agricultural Fields: A Comparative Analysis under Pesticide Stress and Non-Stress Conditions**

^1^Saurabh Gangola, ^1^Samiksha Joshi*, ^2^Geeta Bhandari*, ^3^Garima Pant, ^4^Kahkashan Perveen, ^4^Najat A. Bukhari, ^5^Ranjana Rani, ^6^Anita Sharma*

^1^School of Agriculture, Graphic Era Hill University, Bhimtal, 263132, India

^2^Department of Biosciences, Swami Rama Himalayan University, Dehradun, 248140, India

^3^Department of PDP, Graphic Era Hill University, Bhimtal, 263132, India

^4^Department of Botany & Microbiology, College of Science, King Saud University, Riyadh-11495, Saudi Arabia

^5^School of Agriculture & Food Science, University of Queensland, Gatton Campus, Australia,

^6^Department of Microbiology, GBPUAT, Pantnagar, 263145, India

Corresponding author:

Samiksha Joshi: [joshisamiksha14@gmail.com](mailto:joshisamiksha14@gmail.com)

Geeta Bhandari: [geet33n@gmail.com](mailto:geet33n@gmail.com)

Anita Sharma: [bbmas1975@gmail.com](mailto:bbmas1975@gmail.com)

**(A)**

**(B)**

**Figure: S** Pie chart represents the comparative analysis of 2G and 2GC soil samples at phylum level. A represents the 2G soil sample and B represents the 2GC soil sample
